# Cooperation and Interplay between EGFR Signalling and Extracellular Vesicle Biogenesis in Cancer

**DOI:** 10.3390/cells9122639

**Published:** 2020-12-08

**Authors:** Laura C. Zanetti-Domingues, Scott E. Bonner, R. Sumanth Iyer, Marisa L. Martin-Fernandez, Veronica Huber

**Affiliations:** 1 Rutherford Appleton Laboratory, Central Laser Facility, Research Complex at Harwell, Didcot OX11 0FA, UK; sumanth.iyer@stfc.ac.uk (R.S.I.); marisa.martin-fernandez@stfc.ac.uk (M.L.M.-F.); 2 The Wood Lab, Department of Paediatrics, University of Oxford, Oxford OX1 3QX, UK; scott.bonner@wolfson.ox.ac.uk; 3 Unit of Immunotherapy of Human Tumors, Fondazione IRCCS Istituto Nazionale dei Tumori, 20133 Milan, Italy

**Keywords:** extracellular vesicles (EVs), EV biogenesis, endocytosis, EV heterogeneity, MVB heterogeneity, epidermal growth factor receptor (EGFR), tumour microenvironment, microenvironment subversion, therapy resistance

## Abstract

Epidermal growth factor receptor (EGFR) takes centre stage in carcinogenesis throughout its entire cellular trafficking odyssey. When loaded in extracellular vesicles (EVs), EGFR is one of the key proteins involved in the transfer of information between parental cancer and bystander cells in the tumour microenvironment. To hijack EVs, EGFR needs to play multiple signalling roles in the life cycle of EVs. The receptor is involved in the biogenesis of specific EV subpopulations, it signals as an active cargo, and it can influence the uptake of EVs by recipient cells. EGFR regulates its own inclusion in EVs through feedback loops during disease progression and in response to challenges such as hypoxia, epithelial-to-mesenchymal transition and drugs. Here, we highlight how the spatiotemporal rules that regulate EGFR intracellular function intersect with and influence different EV biogenesis pathways and discuss key regulatory features and interactions of this interplay. We also elaborate on outstanding questions relating to EGFR-driven EV biogenesis and available methods to explore them. This mechanistic understanding will be key to unravelling the functional consequences of direct anti-EGFR targeted and indirect EGFR-impacting cancer therapies on the secretion of pro-tumoural EVs and on their effects on drug resistance and microenvironment subversion.

## 1. Introduction

A crucial step in the evolution of multicellular organisms was the emergence of receptor tyrosine kinases (RTKs). Long considered a prototype of RTK behaviour, epidermal growth factor receptor (EGFR) is of paramount importance to cell function and human health [1]. Indeed, the significance of EGFR as one of the most important receptors regulating growth, survival, proliferation, and differentiation in mammalian cells is underscored by its role in several epithelial cancers, in which EGFR acts as a driver, through overexpression or mutation [2,3]. The regulation of EGFR however, is more complex than initially assumed, and the receptor not only adopts several alternative supramolecular structures [4,5], but also undergoes complex spatial regulation through internalisation and interaction with proteins displayed on various intracellular organelles [6,7].

Derailed endocytosis and cellular trafficking are hallmark features of cancer [8], as they allow cancer cells to manipulate the signalling output [9], traffic receptors to alternate locations such as the nucleus [10,11] and bypass therapy-induced signalling blockages. While it was previously assumed that endocytosis immediately shuts down receptor signalling, there is now a consensus that signalling continues throughout endocytosis, and that the signalling pathways engaged by the endocytosed receptors might be different from those activated at the plasma membrane [12,13,14].

EGFR stands in the centre of carcinogenesis also at the EV level, both as a key membrane protein involved in the transfer of information between parental cancer cells and various bystander cell types in the tumoural microenvironment, and by inducing EV secretion in a variety of contexts.

In this review, we explore how EGFR spatiotemporal regulation intersects with different EV biogenesis pathways, highlighting key regulatory features and interactions, in order to bridge the two fields. We will also emphasize key outstanding questions relating to EGFR-driven EV biogenesis and have a look at available methods to explore them.

## 2. A Preamble to Our Protagonists: EGFR and EVs

### 2.1. EGFR Structures at the Plasma Membrane

**Figure 1:**
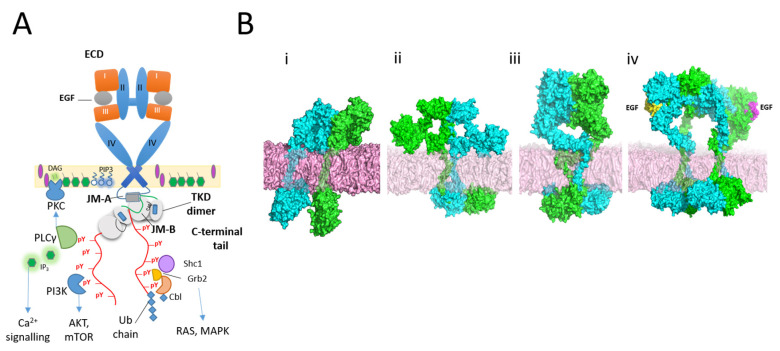
(**A**) Cartoon of EGF-bound EGFR dimer showing the extracellular back-to-back dimer [16,17] and the intracellular asymmetric tyrosine kinase dimer [18]. The latter leads to phosphorylation of C-terminal tyrosines and recruitment of effectors; (**B**) (i–iii) Ligand-free head-to-head, stalk-to-stalk and back-to-back dimers [5]. (iv) Ligand-bound tetramer. The open-ended scheme allows the formation of longer oligomers [4].

Structurally, EGFR is composed of an extracellular domain (ECD), a single-pass transmembrane domain (TMD), and an intracellular module formed by a regulatory juxtamembrane domain (JMD), a catalytic tyrosine kinase domain and a largely unstructured C-terminal tail (CTT). The CTT is where most of the key tyrosine residues reside responsible for scaffolding downstream effectors upon phosphorylation (Figure 1A) [15].

Upon ligand binding, the back-to-back dimer of the extracellular module (ECD) [16,17] promotes across the plasma membrane an asymmetric tyrosine kinase (aTKD) dimer [18] where the activator induces the active conformation of the ATP-binding pocket of the receiver (Figure 1). The aTKD dimer is facilitated by an N-crossing trans-membrane (TM) dimer, an antisymmetric helix dimer of the N-terminal portion of the juxtamembrane domain (JMA) and the positioning of the C-terminal JMB half of the receiver along its interface with the activator [19].

EGFR signalling is initiated at the cell surface through autocatalytic generation of phosphotyrosine residues and consequent recruitment of proteins containing Src homology 2 (SH2) and phosphotyrosine-binding (PTB) domains [1]. This triggers signalling cascades of tyrosine, serine, and threonine phosphorylation events that propagate through the cell interior to elicit specific cell functions [20]. The signal intensity and duration are regulated via the interplay and crosstalk of both the phosphorylation events themselves, and also with E3 ligase dependent ubiquitination, impacting both EGFR and its signalling and trafficking [6].

However, back-to-back dimers do not exhaust the structures present on the cell surface [4,5]. We have previously identified an auto-inhibited ligand-free dimer that coexists with two other kinase-mediated dimers Figure 1B(i–iii), and, more importantly a ligand-bound architecture in which back-to-back dimers assemble oligomers via unoccupied ligand-binding sites Figure 1B(iv). In this architecture, oligomer formation competes directly with ligand binding, and oligomers become less frequent and large with increasing concentrations of ligand, with consequences on assembly and phosphorylation [4].

### 2.2. The Biogenesis of EGFR-Loaded EVs

EGFR-carrying EVs comprise different vesicle subfamilies, which are classified based on their biogenesis and biophysical characteristics. Briefly, the most extensively studied EV subtypes are exosomes, which range in size between roughly 30 nm to 150 nm in diameter, and microvesicles (MVs), sized between roughly 50 nm to 1300 nm in diameter [21,22,23].

Exosomes and microvesicles not only differ in size but also their methods of biogenesis. Exosomes are formed via the endolysosomal pathway while MVs are formed via the outward budding of the cell membrane [24].

**Figure 2:**
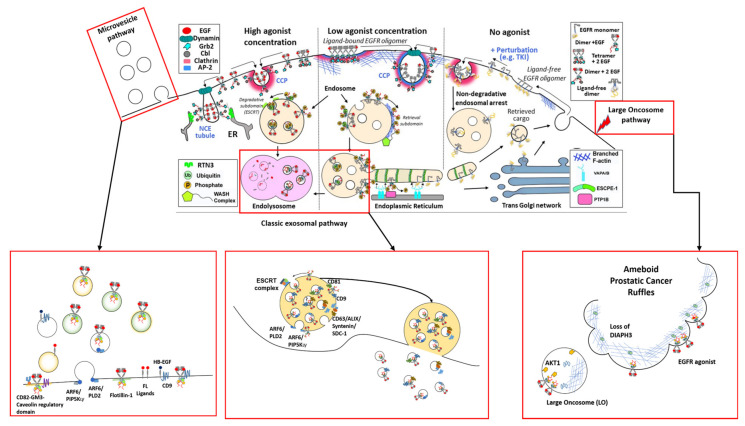
An EGFR-centric view of EV biogenesis. This cartoon describes the possible routes taken by EGFR in its journey through and out of the cell. Top panel: degradative pathway at high EGF dose (left), retrieval at low dose (middle), and recycling (right). Endoplasmic reticulum (ER) contacts with tubular buds, which lead to EGFR dephosphorylation by PTPB1, are also shown. Possible EGFR structures and relevant endocytic effectors are depicted in each location. Red frames highlight the different known pathways of EV biogenesis involving EGFR and its ligands, namely the ARF6-linked microvesicle pathway at the plasma membrane (bottom left), the classical exosome pathway involving sorting at the MVB (bottom middle) and the cancer-specific Large Oncosome pathway (bottom right).

EGFR-loaded exosomes are formed during EGFR endolysosomal trafficking (see Section 3). Briefly, invaginations of the cell membrane containing activated EGFR form membrane bound vacuoles known as early endosomes. Following a series of changes, endosomes mature into late endosomes and subsequently, via the inward budding of the endosomal membrane, membrane-enclosed vesicles called intra-luminal vesicles (ILVs) are formed within the endosome. ILVs are exosomes at their earliest stage. Multiple inward budding events fill endosomes with ILVs; at this stage endosomes become referred to as multivesicular bodies (MVBs) [25] (Figure 2).

In the endolysosomal pathway, MVBs displaying specific surface proteins, including EGFR, fuse with lysosomes resulting in the degradation of ILV contents (Figure 2). Other proteins displayed on MVBs which mark them for lysosomal degradation include the GTPase RAS-related protein RAB7A, the HSP70−HSP90 organizing protein (HOP) complexes, and members of the membrane-fusion soluble *N*-ethylmaleimide-sensitive factor attachment protein receptor (SNARE) complex, including vesicle-associated membrane protein 7 (VAMP7), syntaxin 7 and 8 (STX7/8) [26,27].

On the other hand, MVBs required for the formation of exosomes are transported along microtubules to the plasma membrane. From here RABs, actin and SNARE proteins mediate fusion of MVBs with the cell membrane, and the subsequent release of ILVs into the extracellular space. At this point ILVs become referred to as exosomes [28].

## 3. The Ins and Outs of EGFR Spatial Regulation of EV Biogenesis

The first step in the biogenesis of EGFR-loaded EVs involves endocytosis of ligand-activated EGFR from the cell surface. Once inside the cell, EGFR is sorted into specialized compartments that direct EGFR towards degradation, recycling, or release from the cells loaded in the limiting membrane of EVs. The following subsections describe this process in greater detail (Figure 2).

### 3.1. Entering ILVs Via Ligand-Induced Endocytosis

Under physiological conditions, the activity of EGFR at the plasma membrane is tightly controlled by the interplay between phosphorylation and ubiquitination events, which together ensure timely activation and deactivation of downstream signalling pathways. Soluble high-affinity ligands, like EGF, induce phosphorylation of EGFR at low picomolar range but not ubiquitination [29,30]. While EGFR phosphorylation continues to increase with increasing EGF levels, ubiquitination displays a ”threshold” behaviour best represented in the form of a S-shaped or sigmoidal curve. Studies performed using cell lines have shown that low (<1 ng/mL) concentrations of EGF do not lead to ubiquitination of EGFR, but instead promote clathrin-mediated endocytosis (CME) and recycling. This effect was also observed in vivo where local EGF concentrations are in low nanomolar range [31]. At high concentrations of EGF (>10 ng/mL), CME is still active, but a significant proportion of EGFR instead undergoes non-clathrin endocytosis (NCE) towards the MVB and degradation [29,30]. It is believed that CME becomes saturated at high EGF concentrations prompting a switch to NCE, and this mechanism protects the cells from hyperactivation of EGFR. Striking differences in receptor fate have been noted with other EGFR ligands. For example, transforming growth factor alpha (TGF-α) mainly induces recycling of the receptor and betacellulin (BTC) induces only degradation [32].

Ubiquitination and phosphorylation show cooperativity at physiological levels of the receptor but become uncoupled when EGFR is overexpressed or the concentration of ligand exceeds normal levels [33]. Ubiquitination requires phosphorylation of EGFR at Y1045 and Y1068 (or Y1086) residues as these sites recruit the E3 ubiquitin ligase Cbl and adaptor protein Grb2 respectively to EGFR leading to the formation of K63-linked ubiquitin chains on EGFR [30,34]. Mass spectrometry analysis revealed that roughly 50% of all ubiquitin on EGFR exists in the form of mono-ubiquitin and ~40% in the form of K63-linked poly-ubiquitin chains. K48-linked chains which target substrates for proteasomal degradation only accounted for ~6% of all chains suggesting that K63-linked chains play a far greater role in the regulation of EGFR signalling. Interestingly, it was observed that ubiquitination is crucial for trafficking to lysosomes for degradation, but not necessary for internalization of the receptor [35]. Instead, the E3 ligase activity of Cbl was the most important factor required for internalization and NCE. It is currently unclear what other proteins Cbl might regulate to promote internalization and NCE of EGFR. It is also possible that phosphorylated Y1045 recruits a yet unidentified protein that is responsible for internalization of EGFR as a Y1045F mutant only undergoes CME even at high EGF concentrations [29]. Some NCE pathways have not been studied in depth yet, and an understanding of the factors influencing the choice of carrier, beyond the linkage between ubiquitination and the CME/NCE divide, is lacking. Recently, Caldieri et al. reported a novel NCE pathway that relies on local calcium signalling, and contact sites formed between the endoplasmic reticulum-cell membrane by Reticulon-3 [36].

Besides serving as a discriminant for the early decision points in endocytosis at the plasma membrane, ubiquitination and phosphorylation also drive sorting decisions throughout the endosomal network, down to the crucial split between lysosomal degradation, or retrieval and recycling back to the cell surface [37,38]. Ubiquitinated EGFR is recognised by the by ESCRT-0 and ESCRT-I subunits of the endosomal sorting complexes required for transport (ESCRT-0–III) complexes [39,40]. These multi-protein assemblies serve as molecular machines that recognise the ubiquitin moiety on membrane proteins, like EGFR, and cluster receptors into clearly defined sub-domains of the limiting endosomal membrane (referred to as “degradative sub-domains”) [41]. From these sub-domains, ESCRTs drive receptor inclusion into ILVs that bud into the lumen of maturing endosomes leading to the formation of the LEs/MVBs (Figure 2). The fusion of MVBs with lysosomes leads to the eventual transfer of EGFR to this degradative compartment. A more detailed description of this process has been reviewed elsewhere [37].

In the absence of ubiquitin, EGFR avoids the ESCRT pathway and is instead sorted to and clustered on the limiting endosomal membrane in “retrieval sub-domains”, which are spatially segregated from the degradative sub-domains [42]. From the retrieval sub-domain emerge and mature cytosolic facing tubular buds which lead to the formation of EGFR^+^ transport carriers that interact with the endoplasmic reticulum (ER) and present the EGFR to the ER resident tyrosine phosphatase PTP1B [43,44]. Once the receptor is dephosphorylated, EGFR^+^ carriers recycle the receptor to the cell surface (Figure 2). This spatial segregation of phosphatase activity from the cell surface, triggered by receptor kinase activity, provides temporal and spatial resolution of signalling realised through the endosomal sorting pathway [45,46]. It is as of yet unknown what role, if any, is played by the different oligomeric structures of EGFR in mechanisms of endocytic pathway selection or in directing downstream fate.

Some cancer associated EGFR mutants that display increased auto-phosphorylation and downstream signalling [47,48], and a different structure at the plasma membrane [4,5], are known to be defective in Cbl binding and are not ubiquitinated normally. These mutants display an increased rate of internalization by CME associated with recycling. Blockade of CME shunts EGFR towards micropinocytosis, a clathrin- and dynamin-independent pathway that causes loss of EGFR signalling and receptor degradation and is associated with increased cell death in vitro [49]. A number of E3 ubiquitin ligases apart from Cbl have also been shown to regulate expression of mutant EGFR in various cancer types, some of which target EGFR for degradation by the proteasome. These include CHIP [50], CGRRF1 [51], HUWE1 [52] and SMURF2 [53]. Ubiquitination of EGFR is also regulated by a class of enzymes called deubiquitinating enzymes (DUBs), which remove ubiquitin from EGFR, thereby protecting EGFR from proteolysis. This is one of the many mechanisms that cancer cells exploit to evade cell death thereby conferring resistance to therapy. Examples of EGFR DUBs include USP8 [54], USP17 [55] and USP22 [56].

At this stage, it is important to emphasize that EGFR signalling starts at the plasma membrane but continues to propagate in the endosomes until it is degraded in the lysosome or dephosphorylated by phosphatases such as PTP1B in the ER. Endosomal signalling of EGFR has also been shown to promote the activation of survival pathways [49]. However, excessive EGFR signalling from the endosomes, at least in cell lines that tremendously overexpress EGFR, triggers a pro-apoptotic signalling pathway [57]. These discrepancies might derive from the different cell model and concentration of ligand used in different studies. CME seems to be required for the activation of certain signalling pathways, such as DNA synthesis [58], and in some studies its initiation enhances PLCγ signalling, and inhibits PI3K and MAPK signalling [59]. However other groups have reported that signalling happens primarily at the plasma membrane, both in terms of phosphorylation of MAPK and AKT [60] and in terms of induction of transcriptional response [61]. Interestingly, while Grb2 seems to travel with EGFR in endocytic pits [62,63], it has been reported that HRas does not, providing an explanation from the reduction in EGFR signalling to the MAPK pathway during endocytosis [64].

### 3.2. Exiting the Cell as EV Cargo

Exosomal formation in MVBs can occur either dependent of or independent of the ESCRT-0–III machinery [39,40]. ESCRT-dependent exosomal formation also involves ubiquitination of proteins for selective packaging into ILVs (Figure 2).

ESCRT-independent exosomal formation first involves the hydrolysis of sphingomyelin by neutral sphingomyelinase (N-SMase) to form ceramide [65]. Ceramide allows the generation of membrane subdomains which causes the membrane to become curved and elicits sorting of some cargoes into ILVs [66,67]. Additionally, ADP ribosylation factor 6 (ARF6) has been shown to be involved in ILV budding in a phospholipase D2 (PLD2) dependent manner, as well as in late endosomal sorting of EGFR/HER1, and subsequent trafficking of EGFR to lysosomes for degradation [68]. Furthermore, tetraspanins family members, CD9, CD63 and CD81, all of which are highly enriched on the surface of exosomes have also been shown to regulate ESCRT-independent endosomal sorting [69,70,71,72]. Tetraspanins aid ILV biogenesis either in a similar manner to ceramide dependent biogenesis, via the formation of clusters of these tetraspanins into microdomains with other tetraspanins, transmembrane proteins and cytosolic proteins that bud from the MVB surface [73], or via formation of CD81 rich domains which induce inward budding [74]. Syntenin 1 has also been shown to be involved in targeting of exosome cargo to endosomal membranes for packaging into ILVs [75].

The use of either of these mechanisms can vary between tissues and cell types, however it remains unclear whether these mechanisms can occur simultaneously in the same tissue or cell type, or whether different exosomal subpopulations exist based on these separate formation mechanisms.

In contrast, MV formation occurs independent of the endolysosomal pathway and MVBs, via direct budding of vesicles from the plasma membrane, though does continue to take advantage of the ESCRT machinery. During this process ARF6 and RHO family GTPases rearrange the actin cytoskeleton to initiate membrane budding [76,77]. Following this, the ESCRT-I associated protein, tumour susceptibility gene 101 (TSG101), interacts with ALG-2 associated protein X (ALIX) to mediate sorting of cargoes into MVs and MV release [78]. Both neutral (N-SMase) and acid sphingomyelinase (A-SMase) have also been shown to modulate downstream activation of receptors that trigger MV release [65,79].

### 3.3. EGFR Spatial Regulation Intersects with EV Biogenesis

It has been long known that EGFR signalling promotes the loading of phosphorylated EGFR as cargo in sEVs [80], as well as the release of EVs of different sizes and likely origins (see for example [23,81,82]). Additionally, EGFR signalling can also up-regulate the general internalisation rate of cells by promoting micropinocytosis [83,84], which favours EV uptake [85]. All of these findings hint at a strong linkage between EGFR signalling and EV processing, and this is underpinned by a series of key interactions between EGFR and proteins involved in endocytosis, MVB biogenesis and EV biogenesis (Figure 2). Given this, it is worth considering how the idiosyncrasies of EGFR family endocytosis and signalling might influence the secretion of receptor-positive vesicles or in general influence EV biogenesis pathways and EGFR-dependent EV-mediated intra-cellular signalling.

Regardless of the endocytosis mechanism, EGFR molecules have been found to be clustered in MVB/ILV membranes and are capable of signalling to the MAPK pathway on the limiting membrane of MVBs at this stage [86,87]. EGFR molecules are transported to this compartment only if ubiquitination [88] and kinase activity [89] are preserved. Here, they can influence their own downstream fate.

In fact, EGFR has been shown to induce inward vesiculation of the MVB in combination with Annexin1 [90], a protein which specifically associates with it during internalization [91]. Annexin1 is also involved in the regulation of earlier steps of EGFR endocytosis [92] and is specifically phosphorylated by EGFR at the MVB [93]. Additionally, EGFR signalling is able to stabilize the late-arriving components of the ESCRT complex at the MVB limiting membrane, to accelerate the formation of ILVs and of its own sequestration [94]. There is evidence that EGFR signalling induces the Hrs-dependent formation of a specific subclass of ILVs, characterized by a larger diameter, and that this mechanism is in competition with the formation of CD63-dependent ILVs [95]. The formation of EGFR^+^ ILVs also depend on interactions between the limiting membrane of the MVB and the ER, mediated by Annexin1 and its partner S100A11, which are also involved in cholesterol transport between the MVB and the ER [44]. These contact sites bring the ER phosphatase PTPB1 in contact with its substrates, EGFR and ESCRT0 components [43,96], for de-phosphorylation. Interestingly, Annexin1 has been identified as part of a functional signature associated with aggressiveness in brain tumour-derived sEVs, with expression increased from low-grade to high-grade malignancies, perhaps due to its role in sorting signalling complexes into the vesicles [97].

As explained above (see Section 2.1), EGFR is involved in the regulation of its own inclusion in MVBs through ARF6 and its downstream target PLD2, both of which are required for ILV budding and late endosomal sorting and lysosomal trafficking of EGFR [68]. ARF6 is also involved in the activation of endosomal type Iγ PIP 5-Kinase, another lipid-modifying enzyme, which generates PtdIns4,5P_2_ at the endosomal membrane and controls the incorporation of EGFR in ILVs [98]. ARF6 and PLD2 also promote MV shedding through a MAPK and myosin light chain kinase (MLCK) dependent pathway [76]. Indeed, ARF6 plays a role in EGFR-driven tumorigenesis, where it acts downstream of EGFR and GEP100 to induce invasion and malignancy in breast cancer [99], EMT in head and neck squamous cell carcinoma (HNSCC) [100] and invasion and prognosis in NSCLC [101]. In this line it could be speculated that these effects derive not just from pro-migratory effects in the parental cell, but also display a component of deregulated EV biogenesis contributing to cancer dissemination, but further investigations would be required to confirm it.

**Figure 3:**
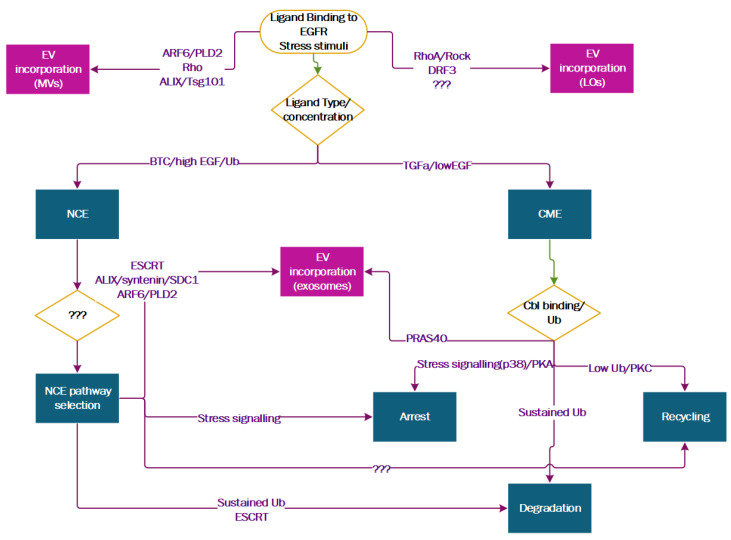
Pathway diagram of EGFR post-endocytic fate.

The nature of the ligand bound to EGFR at the plasma membrane influences the structure of the receptor [102,103,104,105] and the signalling pathways activated downstream of it [106,107,108], determining, together with other cellular factors the fate of EGFR receptors [32,109] (see Figure 3 for a visual summary of EGFR post-endocytic fate).

Therefore, it is not surprising that in certain contexts, different EGFR ligands exert different effects on the EGFR-mediated biogenesis of sEVs. In human keratinocytes, TGF-α is able to induce the release of sEVs, while EGF seems to be less effective (even though reports on the matter are conflicting [82]). Proteomics studies and pathway analysis have identified a proline-rich Akt substrate of 40 kDa (PRAS40) as the master switch that controls sEV secretion in response to EGFR activation by TGF-α [110]. Hopefully, future studies will address whether these differences are a shared mechanism across cell types or are cell-type dependent.

Finally, single-molecule pulldown experiments have shown that the protein-protein interaction pattern of EGFR purified from sEVs is remarkably similar to that of cellular EGFR, indicating that signalling function is preserved [111], while other studies have indicated that the distribution of some phosphorylated isoforms is asymmetric between parental cells and sEVs [112], hinting at yet-unknown, but specific, selection and enrichment mechanisms and cautioning researchers against considering sEV phosphorylation patterns as “representative” of cellular status. Further studies will be required to discern whether the selection of EGFR phosphorylation isoforms for EV secretion is determined solely by their relative abundance in parental cells, or whether there are active selection mechanisms at play.

### 3.4. The Regulators of EGFR Endocytosis Are EV Markers and Regulate EV Biogenesis

A host of other proteins involved in the regulation of EGFR endocytosis and signalling are also regulators or markers of sEV biogenesis pathways, chiefly tetraspanins and lipid-domain organising proteins like flotillins and caveolins. In this section, we will have a closer look at their functional and physical interactions and examine how they may influence EV cargo selection in response to EGFR signalling.

#### 3.4.1. EGFR and Tetraspanins

EGFR and its ligands were found to associate in particular with two EV-relevant tetraspanins, CD9 and CD82, which act as regulators and integrators of its signalling, but might also contribute to its enrichment in EVs derived from cancer cells. EGFR ligands HB-EGF and AREG associate in their full-length, transmembrane forms with CD9 at the membrane of epithelial cells, where the presence of this tetraspanin potentiates their signalling in a juxtacrine context [113,114]. HB-EGF and CD9 can also form a ternary complex with integrins [115], a phenomenon potentially contributing to the cross-talk between these two signalling pathways. CD9 is also physically associated with EGFR at the plasma membrane and can act as a negative regulator of its signalling, by causing its endocytic down-regulation [116]. CD9 is not just a convenient marker of sEVs, but has been found to have a pivotal role in the selection of some types of cargo, such as MHC II, for which incorporation in CD9^+^ microdomains is essential for loading in sEVs [71]. Additionally, there is evidence that CD9^+^ EVs might be a separate population from CD63^+^/ALIX^+^/Syntenin^+^/SDC1^+^ sEVs, a subclass of EVs that is thought to form independently of at least some ESCRT subunits [117]. EGFR itself is found preferentially on CD9^+^/CD81^+^ vesicles [118,119], though other studies have found it in CD9^+^/CD63^+^ vesicles in response to TKI [112]. This finding could provide additional clues on the processes of the complex selection for EV internalization in relation to drug treatments, however further evidence is necessary to unravel how CD9 interactions and other cargo selection mechanisms interplay in EGFR signalling and secretion.

Like CD9, CD82 can also attenuate EGFR signalling [120] and is involved in the PKC-dependent regulation of EGFR trafficking from the plasma membrane via a complex that includes Caveolin-1 and ganglioside GM3 [121,122,123]. The CD82/GM3 complex is also involved in suppressing cell migration induced by EGFR or c-Met [124]. Depletion of CD82 or its regulator TI-VAMP disrupts EGFR trafficking to microdomains and enhances EGFR endocytosis through the CME pathway [122]. Additionally, CD82 has been shown to directly regulate the level of ubiquitination of activated EGFR [123]. All of these findings position CD82 as an important regulator of the EGFR signalling pathway. In glioblastoma, the expression of constitutively activated EGFRvIII has been shown to influence the protein content of exosomes released in the cell culture medium, even if the receptor itself is packaged only in a fraction of them [125]. Interestingly, the researchers noted an antagonistic interaction between EGFRvIII and CD82, which is greatly downregulated in those conditions [125]. Even more interesting, mutant EGFR-induced downregulation of CD82 has been reported also for NSCLC, where the cellular downregulation of CD82 is accompanied by its expulsion through the release of sEVs [126]. Further studies will be needed to untangle the cross-talk between EGFR signalling, tetraspanin domain organisation and EV biogenesis.

#### 3.4.2. EGFR and Lipid-Domain Organizing Proteins

During its signalling journey from the plasma membrane to endocytic compartments and beyond, EGFR interacts functionally and physically with two major classes of lipid-domain organising proteins: flotillins and caveolins. Both of these classes of proteins have been found to regulate EGFR signalling and turnover, and potentially to regulate its inclusion in EVs or be regulated by it at this level. Flotillins are a class of lipid microdomain-associated proteins, which are able to self-assemble into oligomers and form caveolae-like invaginations [127]. Flotillins, are known to internalise in response to EGFR activation [128,129], but, owing to their function as MAPK scaffolds [130], they might be a passenger of EGFR endocytosis more than a driver. In fact, other studies have found that repression of flotillin-1 expression does not affect EGFR internalisation, but rather its oligomerization [131]. There is, however, some evidence that flotillins might regulate the levels of EGFR at the membrane and mediate its cross-talk with E-cadherin signalling [132,133]. Flotillins, in particular Flotillin-1, are often found on sEV limiting membranes, to the point that several authors have used them as a marker of sEV fractions, even though there is evidence demonstrating that Flotillin1^+^ sEVs are a separate population from CD63^+^/ALIX^+^/Syntenin^+^/SDC1^+^ sEVs [75,117]. However, it is still not entirely clear whether CD9 and flotillin-1 are expressed on the same sEV sub-population or are markers of separate CD63-independent sEV subpopulations. This is quite relevant to the field of EGFR research, as both EGFR and its glioblastoma mutant EGFRvIII can be found on Flotillin1^+^ sEVs extracted from the blood of glioblastoma and ovarian cancer patients [23,119], as well as on CD9^+^/CD81^+^ sEVs as mentioned above.

It is worth noting, however, that EGFR can interact functionally with the syntenin-syndecan complex, as well as with ALIX [134,135]. Syntenin-1, a syndecan-binding protein, in fact appears to be co-expressed with EGFR and to regulate its signalling in both glioblastoma [136] and urothelial cell carcinoma [136]. In spite of this, it is not clear whether these interactions are involved in EGFR inclusion in EVs or can have an effect on the inclusion of other proteins in EVs following EGFR signalling.

Much like flotillins, caveolins are an essential family of lipid-microdomain-associated, self-oligomerising proteins which associate with companion proteins called cavins and cholesterol to form plasma membrane invaginations known as caveolae, which perform pleiotropic functions in lipid homeostasis, mechanical stress sensing and membrane organization [137]. It has long been known that inactive EGFR can associate with caveolin^+^ microdomains [138], and that EGFR can phosphorylate caveolin-1 [139] and downregulate its expression [140]. However, as for Flotillin-1, it is still uncertain whether caveolin is involved in EGFR endocytosis or only in signalling organisation and regulation. In fact, an imaging study indicated that caveolin is dispensable in clathrin-independent internalisation of EGFR but is involved in its intracellular trafficking at later stages [141]. Caveolin has been found in sEVs secreted from various solid tumours [142] and in prostatic large oncosomess [143] and in both cases it seems to perform pro-tumour and pro-metastatic functions. Its involvement in EV biogenesis or at least cargo selection is shown by the fact that caveolin-1^+^ EVs secreted from breast cancer cell line MDA-MB-231 have a specific complement of adhesion molecules and a specific role in promoting bystander cell migration and invasion [144]. Additionally, caveolin-1 plays a pivotal role in the alternate endocytic pathways employed by EGFR upon crosstalk with stress signals and is intimately involved in EGFR signalling and the induction of protective autophagy upon hypoxia [145]. Caveolin, in fact, is a direct transcriptional target of HIF1 and HIF2 and its expression in response to an oxygen supply crisis causes clustering of EGFR in caveolar pits and triggers ligand-independent EGFR signalling [146]. Coherently with this, caveolin-1 is enriched in sEVs derived from glioblastoma cell lines under hypoxic conditions [147]. Caveolin-1 is also among the proteins enriched in sEVs derived from EGFRvIII overexpressing glioblastoma cells [125], highlighting its role as a mediator of non-canonical EGFR signalling and as a stress survival mechanism.

Interestingly, a study on the role of lipid microdomains on EV biogenesis found that there is a degree of cross-talk between these two classes of proteins in regard to EV packaging: silencing of flotillin-1 and flotillin-2 had a regulatory role on the incorporation of caveolin. Flotillin-1 seemed to promote caveolin-1 incorporation in sEVs, while flotillin-2 seemed to discourage it. In parallel, pharmacological repression of the synthesis of glucosylceramide led to an increase of caveolin-1 incorporation in sEVs [148]. It is still unclear whether this has any bearing on EGFR secretion in EVs, but there is certainly a lot more ground to cover regarding the interaction between EGFR signalling, lipid microdomain organisation and EV biogenesis.

Finally, sortilin, a transmembrane protein of the VPS10 family, is involved in the sorting of EGFR into endocytic compartments following ligand stimulation as well as in the internalisation and recycling of unliganded receptors, a function that is linked to the limitation of signalling in cancer cells [149]. As well as participating in the spatiotemporal regulation of EGFR, sortilin has also been found on exosomes purified from the A549 NSCLC cell line, in association with EGFR and TrkB. Together these proteins form a trimeric signalling complex involved in exosome release and in the induction of downstream pro-angiogenic signalling [150].

Given the complexity of the interactions that link EGFR with many other actors in the EV biogenesis pathway, it is not surprising that there is still a lack of understanding on many critical factors necessary to dissect which EGFR complexes are selected for MVB-based or plasma membrane-based sEV secretion and based on which signalling flags. It is also unclear whether the fate of EGFR complexes is decided at the plasma membrane level or at later stages, as well as on other important aspects of the interplay between EGFR signalling and all things EV.

## 4. Effects of EGFR-Induced EV Secretion on Cancer Progression

As discussed in the section above, EGFR might have a role not just in regulating its own incorporation in EV compartments, but also that of other proteins involved in its signalling network, particularly in cancer, where it is deregulated. In this section, we observe evidence that this is actually the case, highlighting the cancer histotypes in which it occurs, the processes unleashed and the EGFR signalling partners involved (Table 1).

### 4.1. Head and Neck Cancer, EGFR-Induced EMT and EV Secretion

Head and neck carcinomas are a rather frequent and heterogeneous type of cancer that develop from the epithelia of the oropharyngeal cavity, nose and upper respiratory tract following exposure to risk factors such as tobacco smoke, alcohol or HPV infection. A subset of these cancers progress due to the presence of somatic mutations in the EGFR gene and are associated with significantly worse prognosis [158].

Epithelial-to-mesenchymal transition (EMT) is the reactivation of pathways involved in embryonal development and wound healing which lend enhanced growth and aggressiveness, as well as drug resistance, to cancer cells. This process represents a key feature of the pathogenesis of head and neck cancers [159] and is a significant prognostic factor in meta-analysis [160]. Owing to its role in epithelial wound healing [161], EGFR is a key player in the induction of EMT (see for example [162,163]), as are EVs (reviewed in [164]), and the two appear to be strictly linked in renal wound healing [165]. Because of this, it is perhaps unsurprising that EGFR-mediated EV biogenesis in HNC appears to be linked to EMT.

In EGFR-overexpressing HNC cell line A431, simultaneous stimulation of EGFR with TGF-α and blockade of E-Cadherin induces EMT. EGFR is sequestered away from the membrane, alongside Tissue Factor (TF), a protein involved in the coagulation cascade, and this is accompanied by a surge in sEV release, where the levels of EGFR do not incur major changes, but TF is highly enriched [151]. This is particularly relevant for the connection between EGFR signalling in parental cancer cells and the induction of angiogenesis through dysregulated coagulation, a process which occurs also in glioblastomas, where it is initiated by EGFRvIII and proceeds though a TF feedback loop [166].

Besides the induction of TF enrichment, major changes in the overall proteome of EVs derived from A431 cells have also been registered as a consequence of EGFR-mediated EMT induction, with an increase in proteins involved in cellular growth, cell-to-cell signalling, and cell movement, including integrin α2 and CD9 [152].

The link between EGFR signalling, EMT and EV secretion is readily apparent in Oral Squamous Cell Carcinoma (OSCC) cell line HSC-3, where exposure to EGF induces both EMT and an enrichment of EGFR in the EVs secreted by the cancer cells [153]. These EGFR^+^ EVs, in turn are able to induce EMT markers in bystander cells [82]. This chain of events can also be initiated by nicotine upstream of EGFR secretion [153] and is enhanced in metastatic cell sublines [167].

In conclusion, EGFR-mediated EV biogenesis seems strictly wedded to the initiation of EMT and cancer progression in various HNC subtypes.

### 4.2. Ameboid Prostate Cancer and the Release of LOs

Prostatic carcinoma is another histotype in which EGFR has been found to have a pivotal role, specifically in progression and resistance to androgen suppression therapy (see for example [168,169,170]).

Ameboid prostate cancer, a particularly aggressive and invasive tumour subtype, displays a specific mode of cell migration mediated by RhoA/Rock, which is accompanied by extensive non-apopototic plasma membrane blebbing [171]. This cancer type also releases exceptionally large (>1 μm in diameter) plasma membrane-derived EVs called Large Oncosomes (LOs) which are marked by caveolin-1 and contain ARF6, c-Src and Akt proteins as well as metalloproteases [81,143]. Blebbing, migration and LO shedding are correlated and can be induced by EGFR overexpression, overexpression of EGFR ligands, or by HB-EGF derived from prostatic stromal cells [81,154]. These processes are further regulated by actin nucleating protein DIAPH3, a frequent target of mutations or deletions in prostate cancer, whose loss is associated with enhanced shedding and an increase in migratory potential [81,143]. Interestingly, DIAPH3 loss is associated not only with alterations in the cytoskeleton, but also with endosomal accumulation of EGFR, with prolonged signalling and hyperactivation of the EGFR/MEK/ERK signalling axis, hinting again at a pivotal role of EGFR in this process [172].

LOs are readily taken up by bystander cells and are able to simultaneously activate EGFR and Akt signalling in recipient stromal cells, reprogramming them towards pro-tumoural support [81]. They also repress the proliferation of monocytes and macrophages via inhibition of Akt-1 by miR-125a, conferring a double advantage to the ameboid cells [154]. Proteomics studies have confirmed that LOs are enriched with a different complement of proteins from smaller EVs, many of which are involved in cancer progression and resistance to therapy [173].

### 4.3. Glioblastoma and EGFR as a Regulator of EV Packaging

Glioblastoma is the most common primary brain tumour and in spite of considerable efforts, its prognosis is still very unfavourable. EGFR signalling and mutations play a key role in its pathogenesis [174], as does sEV-mediated communication [175]. Microenvironmental subversion is a key feature of its pathogenesis and progression [176] and tumour-derived sEVs have been shown to reprogram the main constituent of their microenvironment, astrocytes, to serve in supporting role for tumour growth [177].

Since the first seminal papers [23,178] there has been a large research focus on the role of EGFR expressed on glioblastoma-derived EVs and on its ability to be transferred to bystander cells and influence the microenvironment, however the role of this receptor in glioblastoma is not limited to being a form of cargo. Overexpression of EGFRvIII, a truncation mutant of EGFR which is activated in absence of ligand [179], in glioblastoma cells has been shown to radically alter the regulation of sEV biogenesis and sEV contents, favouring the secretion of pro-invasive proteins such as CD44 [125].

The role of EGFR in miRNA maturation [180] through its interactions with AGO2 [181] opens another interesting avenue of investigation, as EGFR or EGFRvIII amplification in glioblastoma is associated with higher grade [97], which in turn is associated with a specific pattern of sEV-packaged miRNAs regulating pro-tumoural functions in the microenvironment [182].

These findings hint at a master role for EGFR in the subversion of the tumour microenvironment in glioblastoma which warrants further investigation.

### 4.4. EGFR-Driven Cancer Cell Metabolism Impacts EV Formation

In the 1920s Otto Warburg observed that tumour cells took up high rates of glucose to convert it to lactate despite the presence of oxygen, the so called “Warburg effect”, instead of the more effective oxidisation of pyruvate in the mitochondria (oxidative phosphorylation, OXPHOS) to generate ATP. This allows cancer cells to recycle NADH to NAD^+^ leading to the loss of carbon during lactate release, at the same time providing a pool of glycolytic intermediates, which favour biosynthetic pathways branching from glycosysis. Ongoing research however showed that tumours also consume oxygen to oxidize pyruvate in the mitochondria, indicating that both phenomena can occur contemporaneously, or even that some cancer cells are dependent of OXPHOS [183]. Oncogenic signalling reprograms the metabolism by shifting tumour cells to high glycolysis and lactate production, which, once extruded into the tumour microenvironment is uptaken by other tumour cells and causes immune suppression, an effect that could be demonstrated also for EGFR. Different metabolic processes ranging from the biosynthesis of fatty acids and pyrimidines to glucose catabolism involve EGFR signalling. This can occur directly through phosphorylation of rate-limiting enzymes or indirectly by activating the transcription factor MYC and the AKT signalling cascade [184].

In 2008 Weihua et al. found that EGFR can promote glucose uptake through physical association and stabilisation of sodium/glucose cotransporter (SGLT1). This is independent from EGFR kinase activity and ensures the maintenance of the basal glucose intracellular level thereby preventing cancer cells from autophagic cell death [185]. Similarly, EGF can accelerate glucose intake and lactate release in breast cancer and in glioma cells, where EGF-mediated nuclear translocation of Pyruvate kinase muscle isozyme M2 (PKM2), a limiting glycolytic enzyme that catalyses the final step in glycolysis, induces tumourigenesis and cell proliferation [186].

Among the pro-tumoral activities of cancer-derived EVs is their potential to modulate glucose metabolism, a phenomenon that may be regulated by proteins of the glycolytic pathways carried by EVs, like PKM2, leading to enhanced cell proliferation and resistance to apoptosis of cancer cells, as shown by experiments with EVs of Triple-Negative breast cancer cell lines displaying high and low levels of glycolytic activity [187]. EVs can contain functional glycolytic enzymes and can also generate ATP themselves depending on their ATPase activity levels, potentially providing the cell with the necessary energy for EV internalisation. For instance, EVs from malignant cells display a lower ATPase activity than EVs from non-malignant cells, leading to the generation of an ATP rich extracellular microenvironment [188]. Elevated levels of extracellular ATP induce inflammation and release of EVs, which in turn contain proteins involved in cell adhesion/extracellular matrix organization, autophagy-lysosomal pathway and cellular metabolism and which may impact the uptake by the target cell, as shown for microglia-derived EVs [189].

Lactate accumulation in the TME is also influenced by TKIs. Indeed, EGFR- and MET-addicted tumour cells undergo a metabolic shift towards increased glycolysis and production of lactate during prolonged treatment with TKIs and onset of resistance. In turn lactate instruct cancer-associated fibroblasts (CAFs) to produce hepatocyte growth factor (HGF), which activates MET and sustains the development of adaptive resistance to TKIs [190]. CAF-derived EVs can also modulate the metabolism of cancer cells through the import of amino acids, lipids, TCA metabolites and mtDNA, leading to increased glycolysis and OXPHOS in the cancer cell [191]. Finally, tumour EVs can induce metabolic alterations in neighbouring tumour or microenvironment cells, as demonstrated for EVs carrying functional GLUT-1 deriving from mutant KRAS colorectal cancer cells, induce glucose uptake, lactate and glutamate production in colonic epithelial cells [192].

Metabolic alterations induced by EGFR signalling appear to govern at least in part the secretion and molecular composition of cancer cell EVs. In turn these EVs can shape the uptaking cell depending on its metabolic state. Additionally, EVs exposing EGFR ligands can influence the metabolic state of their “target cell” by binding to EGFR on the one side, while on the other they can introduce in the uptaking cell a variety of metabolic enzymes and metabolites, such as lactate, pyruvate, monocarboxylate transporters, lactate dehydrogenase (LDH) and other glycolytic enzymes, pyruvate and prostaglandins [193]. Both EGFR and EVs are also involved in fatty acid synthesis and cholesterol metabolism [194] and further studies will be needed to unravel their implications on the cell itself, the neighbouring cells or at distance.

### 4.5. NSCLC, Basal-Like Breast Cancer and the Role of Stress-Induced Endocytosis

EGFR is overexpressed in about 50% of NSCLC patients and has a significant negative prognostic value, while mutations in the TKD which can be targeted with TKIs are present in ~10% of cases [195]. Anti-EGFR therapy has represented a step change in the treatment of these subtypes, however resistance and therapy failure through multiple mechanisms, both EGFR-dependent [196] and EGFR-independent [197,198,199,200,201] are still key issues. EVs have long been recognized as a key tool in a tumour’s therapy resistance arsenal [202,203] and NSCLC is a perfect example of their power in action. In the gefitinib-resistant NSCLC subline PC9-R, which expresses the EGFR T790M (or T766M in alternative numbering system) mutation, sEVs are enriched with components of the Akt/mTOR signalling pathway, which confer to recipient cells not only protection against gefitinib, but also enhanced proliferation and invasion [155]. This mechanism seems shared between NSCLC cell lines that express EGFR T790M [156] and has conceptual parallels in the ability of A431 cells to up-regulate pEGFR and pERK packaging in EVs, as well as EV emission in response to TKI treatment [113]. On the other hand, EGFR is downregulated on the surface of EVs in response to TKI in cell lines which are not dependent on EGFR signalling, such as luminal breast cancer cell line MCF-7 [204].

The microenvironmental response of EGFR-dependent cells to TKI treatment, however, is not limited to compensatory signalling. sEVs secreted by the gefitinib-sensitive parental line PC9 treated with gefitinib are able to decrease the effectiveness of cisplatin treatment in autologous cells. This antagonistic effect is mediated by the induction of cytoprotective autophagy in the recipient cells by sEVs derived from gefitinib-treated cells [155], with autophagy being a known mechanism of antagonistic interactions between the two drugs that allows cells to escape from apoptosis [205]. The induction of cytoprotective autophagy might be related to known mechanisms of endosomal arrest of EGFR induced by therapeutic interventions and other stressors (reviewed in [206]), such as prolonged starvation, UV exposure, cisplatin, TNFα and TKIs [207]. Stressors can induce stabilisation of ligandless EGFR in endosomes, where it interacts with Beclin1-Vps34 and stimulates cytoprotective autophagy through kinase-independent mechanisms [208], some of which are mediated by p38 MAPK signalling, which induces Ser/Thr phosphorylation of the C-terminal tail of EGFR and of Rab5 effectors [209]. TKI-induced internalisation of unphosphorylated EGFR happens also in cells with secondary and tertiary mutations that confer TKI resistance in presence of osimertinib and is mediated by CME [210]. Stress-related endocytosis of EGFR, including by TKIs, is also linked to aberrant signalling from the MVB, mediated by the interaction between the receptor and ESCRT complex components such as Tsg101/ALIX [211]. Similar phenomena of EGFR endosomal arrest can be induced also by cetuximab [212], hypoxia [181] and ionising radiation [213] through a caveolin-dependent pathway activated by c-src and PKC.

Oxidative stress, which can be induced by treatment with TKIs [214], is another inducer of stress-related endocytosis. This stressor induces caveolin-mediated internalisation through a yet-unknown mechanism [206], possibly mediated by a non-canonical EGFR conformation [215].

Finally, cell lines which are resistant to gefitinib display decreased expression of Rab25, a Rab11 family member which controls EGFR recycling, and whose deficit favours anti-apoptotic arrest of EGFR in endosomes [207]. Gefitinib treatment can also induce changes in the levels of immune checkpoint protein PD-L1 in sEVs derived from NSCLC cell lines, which were resistant to different drugs, including gefitinib, erlotinib and crizotinib. The increased expression of this immune checkpoint leads to enhanced immune suppression [216], an effect that can be countered by immune checkpoint blockade.

The expression of PD-L1 is also associated with EGFR and its mutations. Specimens from NSCLC patients show that high levels of PD-L1 are associated with activating EGFR mutations and this is associated with poor patient prognosis. Of note, the level of PD-L1 expression appears to depend on EGFR signalling [217]. PD-L1 can be expressed both by tumour cells and by infiltrating immune cells and the binding of this ligand to its cognate receptor PD-1 (mainly expressed by T cells) leads to the functional inhibition of anti-tumour immune responses. The blockade of the PD-L1/PD-1 axis by anti-PD-1 or anti-PD-L1 antibodies represents one major therapeutic strategy in current cancer clinical practice [218].

EGFR activation or mutation has been demonstrated to lead to the upregulation of PD-L1 expression by NSCLC cells, thereby fostering immune evasion by T cell inhibition, an outcome that could be reversed by TKI inhibition [219]. Since EGFR activation also leads to an increase of EV production and release, EVs harbouring PD-L1 on their surface may contribute to amplifying T cell inhibition on the one side, while on the other they may activate EGFR via their EGFR ligand expression, further potentiating the EGFR-PD-L1 loop. However, investigations conducted in basal-like breast cancer, a subtype also known as Triple-Negative breast cancer, where EGFR is often overexpressed demonstrated that relationship between these phenomena are more complex than previously thought [220]. Breast cancer cells lacking ALIX, a protein involved both in EGFR spatial regulation [134,135] and in EV secretion [133], displayed an enhancement of EGFR activation, as well as an impairment in PD-L1^+^ EV release which conferred a more immunosuppressive phenotype to the tumour cells [157]. It is worth noting that ALIX is an important interaction partner of Tsg101 [221], an ESCRT-I protein which is regulated by EGFR at the MVB level [94]. ALIX and Tsg101 are also involved in the stress-induced endocytosis and intracellular accumulation of EGFR [211]. This suggests that there might be multiple layers of interaction at play in regulating the expression of PD-L1 downstream of EGFR signalling alterations.

In patients the effects of EGFR-TKI treatment on EGFR mutant NSCLC TME were visible in re-biopsies of those progressing during therapy. EGFR-TKI led to increased PD-L1 expression from 14% at baseline to 28% of patients, accompanied by higher tumour mutational burden, a measure of how many mutations per megabase of genome the tumour has managed to accumulate. Of note, progression-free survival was longer for patients exhibiting high PD-L1 expression during subsequent treatment with anti-PD-1 antibodies, indicating that dissecting the impact of EGFR-TKIs on TME may contribute optimizing Immune Checkpoint Inhibition therapy [222].

Not least due to the entanglement of these two pathways, therapies that reverse immune tolerance through the blockade PD-1/PD-L1 and CTLA4 have attracted major interest in clinical trials of NSCLC and other EGFR-related malignancies. Despite only subsets of EGFR mutant tumours displayed responses to immune checkpoint blockade, the understanding of the underlying mechanisms in relation to EGFR and immune checkpoint expression may provide new attractive therapeutic paths [223].

## 5. Perspectives

EGFR plays multiple roles in the life cycle of EVs. It is involved in the biogenesis of specific EV subpopulations, it acts as an active cargo and it can even influence the uptake of EVs by recipient cells.

While the data presented seems to hint to the fact that EGFR signalling, endocytosis and exocytosis are all linked together, with receptor secretion on EVs positioned as a third post-endocytic fate alongside recycling and lysosomal degradation, the specifics of this linkage and the signals leading to this route are a lot less clear.

NCE of EGFR has been proven to be linked with EGFR ubiquitination and transport to the MVB [29,30], however if this is the principal mechanism of “classical” exosome formation, we are still missing some key information on the signals that rescue ubiquitinated EGFR from lysosomal degradation. The role of post-translational modifications (PTMs) is likely to be key, and it has been already demonstrated that some phosphorylated EGFR isoforms are preferentially enriched in EVs compared to the parental cell [112], but a systematic understanding of this “language” is still lacking. In addition to this, regulatory proteins interacting with PTMs on the EGFR receptors and cross-talk with adjacent signalling pathways in the network will act as a major source of regulation and fine-tuning of cellular responses. As for EGFR-regulated EV secretion that does not involve the receptor itself, we are still missing key information about what downstream effectors are impacted by EGFR signalling and their role in the regulation of EV biogenesis processes.

Multidisciplinary studies incorporating proteomics and phospho-proteomics, elegant use of genetic modifications as well as live-cell 3D imaging with high temporal and spatial resolution, 3D super-resolution and possibly CLEM will be required to follow the journey of target proteins through and out of the cell and fully dissect the where, when and how of the selection processes involved in EGFR-mediated EV biogenesis. Improvements in EV labelling and protein tagging will also be required, as discussed in the 2019 ISEV position paper on biological membranes and EV biogenesis [224], as well as an improvement in endocytosis inhibitor selectivity, allowing the distinction between CME and NCE pathways [225].

This mechanistic understanding will be key to unravel the functional consequences of anti-EGFR targeted therapies on the secretion of pro-tumoural EVs and their effects on drug resistance and microenvironment subversion. As liquid biopsies take centre stage as a tool to diagnose and manage cancer [226,227,228], EVs are increasingly considered an attractive way of extending this concept and accessing non-genetic molecular information about solid tumours through minimally invasive means (see for example [229,230]). In this context, identifying EV subclasses, derived from both cancer cells and microenvironment components, linked to defined pathological processes would enable their use as complementary biomarkers to circulating cell-free DNA and circulating tumour cells. In recent years, numerous studies have demonstrated the utility of EVs as cancer biomarkers that permit longitudinal monitoring of tumour heterogeneity and the early identification of cancer subtypes [231,232,233], as well as monitoring of microenvironment subversion [234], tumour progression and prognostic determination [235], and response to therapy in order to tailor therapeutic interventions [236,237]. Further developments in this field will bring us closer to the goal of delivering personalized oncologic care.

## Figures and Tables

**Table 1:** Evidence for EGFR-mediated EV biogenesis in cancer.

Cancer Type	Effect	Citation
HNSCC (A431)	EV surge and TF emission upon EGFR-induced EMT	[151]
HNSCC (A431)	EV proteome shift upon EGFR-induced EMT	[152]
OSCC (HSC-3)	Simultaneous induction of EMT and EGFR enrichment in EV upon EGFR stimulation	[153]
Ameboid Prostatic Carcinoma (DU-145)	EGFR signalling induces Large Oncosome (LO) formation	[81,154]
Glioblastoma	EGFR vIII expression alters EV proteome	[125]
NSCLC (PC9R)	EGFR T790M mutation alters composition of EVs to confer gefitinib resistance to bystander cells	[155]
NSCLC (PC9R, H1975)	EGFR T790M mutation alters EV proteome	[156]
Basal-like breast cancer	EGFR intracellular accumulation and suppression of PD-L1 release in EVs are co-regulated by ALIX	[157]
